# Third-wave interventions for eating disorders in adolescence – systematic review with meta-analysis

**DOI:** 10.1186/s40479-021-00158-6

**Published:** 2021-06-14

**Authors:** Arne Buerger, Timo D. Vloet, Lisa Haber, Julia M. Geissler

**Affiliations:** 1 Department of Child and Adolescent Psychiatry, Psychosomatics and Psychotherapy, University Hospital of Wuerzburg, Margarete-Hoeppel-Platz 1, 97080 Wuerzburg, Germany; 2 German Centre of Prevention Research in Mental Health, University Wuerzburg, University Hospital Wuerzburg, Wuerzburg, Germany

**Keywords:** DBT, Adolescence, Eating disorders, Third-wave psychotherapy, Meta-analysis, Review

## Abstract

**Context:**

Third-wave therapies have demonstrated efficacy as a treatment option for EDs in adulthood. Data on the suitability for EDs in adolescence are lacking.

**Objective:**

To estimate the efficacy of third-wave interventions to reduce ED symptoms in adolescents in randomized controlled trials (RCTs) and uncontrolled studies.

**Data sources:**

We systematically reviewed the databases PubMed (1976-January 2021), PsycINFO (1943-January 2021), and the Cochrane database (1995-January 2021) for English-language articles on third-wave therapies. References were screened for further publications of interest.

**Study selection:**

RCTs and pre-post studies without control group, comprising patients aged 11–21 years (mean age = 15.6 years) with an ED diagnosis (anorexia nervosa, bulimia nervosa, binge eating disorder, eating disorder not otherwise specified) investigating the efficacy of third-wave psychological interventions were included. Efficacy had to be evaluated according to the Eating Disorder Examination or Eating Disorder Examination-Questionnaire, the Eating Disorder Inventory-2, the Eating Disorder Inventory-3, or the Structured Interview for Anorexic and Bulimic Disorders for DSM-IV and ICD-10. The outcome assessed in the meta-analysis was the EDE total score.

**Data extraction:**

Independent extraction of data by two authors according to a pre-specified data extraction sheet and quality indicators.

**Data synthesis:**

We identified 1000 studies after removal of duplicates, assessed the full texts of 48 articles for eligibility, and included 12 studies with a total of 487 participants (female 97.3%/male 2.6%) in the qualitative synthesis and seven studies in the meta-analysis. Articles predominantly reported uncontrolled pre-post trials of low quality, with only two published RCTs. Treatments focused strongly on dialectical behaviour therapy (*n* = 11). We found moderate effects of third-wave therapies on EDE total score interview/questionnaire for all EDs (d = − 0.67; z = − 5.53; CI95% = − 0.83 to − 0.59). Descriptively, the effects appeared to be stronger in patients with BN and BED.

**Conclusion:**

At this stage, it is not feasible to draw conclusions regarding the efficacy of third-wave interventions for the treatment of EDs in adolescence due to the low quality of the empirical evidence. Since almost all of the identified studies used DBT, it is unfortunately not possible to assess other third-wave treatments’ efficacy.

**Supplementary Information:**

The online version contains supplementary material available at 10.1186/s40479-021-00158-6.

## Background

Eating disorders (ED) such as anorexia nervosa (AN) and bulimia nervosa (BN) come with comorbid psychiatric disorders, serious physical complications and a high risk of chronicity and mortality [1, 2]. The Global Burden of Disease study found EDs in adolescence to be the 12th leading cause of disability-adjusted life years in 15–19-year-old girls in high-income countries [3, 4]. It is therefore not surprising that EDs negatively influence socioeconomic achievement [5]. According to our current understanding of the disease mechanisms, EDs share characteristics of emotion dysregulation disorders such as borderline personality disorder (BPD). Patients with EDs often suffer from high levels of aversive tension, especially in social situations and in situations where they are confronted with their body, body weight, or food intake [6]. Similar to patients with BPD, EDs are characterized by high-risk behaviours (e.g. life-threatening weight loss, vomiting, laxative abuse). In addition, regulation of unpleasant emotions appears to be behind both restrictive and bulimic eating behaviour [7].

Despite the severity of EDs, specialist healthcare services for adolescents with EDs are rare in most countries, and clinicians often assess ED therapy as complex [8].

### Anorexia nervosa

International guidelines for AN in adults recommend CBT-based treatment for moderate to severe AN [9–11]. However, the number of studies in adolescents is limited. One uncontrolled study assessing CBT-based day-patient (DP) treatment for AN found significant improvements in terms of ED symptoms and weight restoration [12]. A study by Herpertz-Dahlmann and colleagues indicated that DP treatment after short inpatient care in adolescent patients with non-chronic AN may be equally effective as inpatient treatment (IP) for weight restoration (BMI) and maintenance during the first year after admission [13]. An uncontrolled study by Dalle Grave and colleagues suggested that outpatient “enhanced” cognitive behaviour therapy (CBT-E) is also effective in reducing ED symptoms in adolescence [14]. Family-based therapy (FBT) is the most widely evaluated treatment for AN in children and adolescents. Fisher and colleagues [15] conducted a review with meta-analysis on 25 trials investigating FBT for EDs (*n* = 17 trials including or focusing on adolescents). The authors found no evidence for a superiority of FBT over treatment as usual or other psychological interventions in terms of remission rates or ED pathology. Due to the low quality and the low number of identified studies, the authors concluded that there is insufficient evidence to determine whether certain variations of FBT (e.g. inpatient vs. outpatient) are more effective than others. However, despite the scarcity of research, results indicate that specialized treatments for AN, such as FBT, are no more effective than treatment as usual.

### Bulimia nervosa

Guidelines for EDs in adults recommend CBT, DBT, psychodynamic treatment, interpersonal therapy, FBT, and self-management as effective therapeutic options for BN [9].

In adolescence, family therapy and CBT-A have been indicated to be effective in reducing bulimic behaviour [16, 17]. An uncontrolled study by Dalle Grave et al. [18] applied CBT-E to non-underweight (BMI ≥ 18.5) adolescents with a diagnosis of BN or EDNOS, and found a marked reduction in ED pathology.

Le Grange and colleagues [19] conducted an RCT with BN patients (including subthreshold BN) comparing FBT-BN (focused on parental control) to CBT-A (focused on changing behaviour and cognitions). FBT-BN was more effective than CBT-A in terms of abstinence rates from binge eating and purging behaviour at the end of treatment and at the 6-month follow-up, but not at the 12-month follow-up. An RCT comparing CBT and psychodynamic therapy in female adolescents and young adults found comparable rates of remission for both treatments, with a small advantage for CBT on binging/purging and a small advantage for psychodynamic therapy on eating concern [20].

For BN in adolescence, there is a marked paucity of studies evaluating the efficacy of specialized treatment for EDs.

### Third-wave interventions as a treatment option for EDs

Although substantial progress has been made in specialized treatments of adolescents, there is still room for improvement regarding treatment retention, outcomes, and dropout rates, and in terms of well-conducted RCTs with larger numbers of cases. Furthermore, it can be argued that a broader range of effective ED treatments is needed to improve long-term efficacy, increase levels of treatment acceptability among clinicians and patients, and provide a broader range of evidence-based treatment options or dissemination [21]. The perception and management of emotions, identity and interactional disturbances appear to play an important role in eating disorders, but are not adequately addressed by the currently established second-wave interventions [12].

In this context, third-wave behavioural therapies constitute a potential alternative treatment for EDs [18]. Comprising many of the components of CBT that are effective in adults with EDs (e.g. exposure, self-monitoring), these therapies additionally target the understanding and awareness of cognitions and emotions, and focus on processes such as acceptance, mindfulness, attention, dialectics, therapeutic relationship, and values [22]. CBT and third-wave methods both support adaptive emotion regulation strategies, but target different emotional processing pathways (response-focused vs. antecedent-focused emotion regulation strategies) [23]. This new wave of behaviour therapy is generally agreed to include acceptance and commitment therapy (ACT), compassion-focused therapy (CFT), dialectical behaviour therapy (DBT), mindfulness-based interventions (MBI), and schema therapy (ST). However, a consensus definition of third-wave behaviour therapy is still under discussion [24].

To date, three meta-analyses have analysed the efficacy of third-wave therapies for EDs [25–27]. The meta-analysis (*n* = 9) by Lenz et al. (2014) examined the efficacy of DBT for individuals with EDs and co-occurring depressive symptoms. The main outcomes were the reduction of eating disorder episodes (vomiting, binge eating, starvation) used to dysfunctionally regulate one’s emotions, and the reduction of depressive symptoms. The authors found a large effect on reducing the number of eating disorder episodes in women, as well as medium to large effects on reducing depressive symptoms. Godfrey et al. (2015) included 19 studies in their meta-analysis on mindfulness-based interventions for BED, and found moderate to large effects on binge eating. Linardon et al. (2019) estimated in their meta-analysis (*n* = 12 RCTs) that while efficacy has not yet been empirically demonstrated, due to methodological limitations in the conducted studies (e.g. no follow-up, no comparison with waitlist control group), third-wave treatments nevertheless have the potential to be effective. For the time being, the authors continue to rate CBT in adults as a recommended treatment for BN and BED, and even as a leading treatment for AN. However, high statistical heterogeneity between the studies was cited as a limiting factor in all of the meta-analyses.

None of the meta-analyses included patients under 18 years of age. While this is not uncommon for research on psychotherapeutic treatments in general, it is nevertheless surprising given the peak age incidence of 10–14 years for AN and 15–19 years for BN [2, 28, 29]. Moreover, the age of onset is decreasing [30], further underlining the necessity for treatment options for children and adolescents. Consequently, we ask: 1) Which third-wave treatments have been adapted for EDs in adolescents?, and 2) How effective are third-wave interventions in reducing ED symptoms in adolescents in controlled and uncontrolled studies?

## Methods

We conducted our review with meta-analysis according to the PRISMA guidelines. For the complete PRISMA checklist, see Supplement 1. Eligibility criteria and analysis methods were specified in advance and documented in a protocol (Supplement 2).

### Search protocol and information sources

We searched PubMed (1976-January 2021), PsycINFO (1943-January 2021), and the Cochrane database (1995-January 2021) for English-language articles on third-wave therapies (search terms: “third wave” OR dialectical behavior therapy OR dialectical behaviour therapy OR dialectic behavioral therapy OR dialectic behavioural therapy OR DBT OR mindful* OR acceptance OR schema therapy OR compassio*) combined with ED (search terms: eating disorder OR bulimi* OR anorexi* OR binge OR EDNOS) and adolescence (search terms: adolesc* OR teen* OR youth OR children OR childhood OR pediatric). For details on the search strategies, please refer to Supplement 3. We performed the last search on 15^th^ January 2021. Subsequently, we screened the references in the publications obtained from step 1 for further relevant articles. After removing duplicates, we screened titles and abstracts. If studies were relevant to the topic, we obtained the full texts.

### Eligibility criteria

This review included all studies meeting the PICOS inclusion criteria specified below, published in English in a peer-reviewed journal up until 15 January 2021.

#### Types of participants

We considered samples comprising participants aged 11–21 years with an ED diagnosis (AN, BN, binge eating disorder, EDNOS). If a study sample extended beyond that age range, the publication needed to separately report results for the adolescent subgroup.

#### Types of interventions

This review was limited to studies investigating the efficacy of third-wave psychological interventions, i.e. treatments based on ACT, CFT, DBT, MBI, or ST.

#### Types of comparisons

Presence of a comparison / control group was not required for inclusion in the review. We also considered pre-post studies.

#### Types of outcome measures

Efficacy had to be evaluated according to the Eating Disorder Examination (EDE) or Eating Disorder Examination-Questionnaire (EDE-Q) [31], the Eating Disorder Inventory-2 (EDI-2) [32, 33], the Eating Disorder Inventory-3 (EDI-3), [34] or the Structured Interview for Anorexic and Bulimic Disorders for DSM-IV and ICD-10 (SIAB-EX) [35].

#### Types of studies

We included RCTs and pre-post studies. Exclusion criteria were single case studies, reports on prevention, and non-empirical publications (reviews, theoretical papers).

### Study selection

After removal of duplicates, abstracts were screened by one of the authors (JG) and independently screened by a second researcher (LH) to determine their relevance to this review. Two authors (AB, JG) then independently screened the full text of the remaining articles. Disagreement was resolved through discussion. Studies were included in the meta-analysis if they reported outcomes on the EDE or EDE-Q.

### Data collection process

Data extraction was independently performed by AB and JG on a standardized extraction sheet (based on the Cochrane Consumers and Communication Review Group’s data extraction template) and subsequently discussed and integrated. Discrepancies were resolved through discussion. If no consensus could be reached, a third author (LH) assessed the data. We extracted data on the sample (sample size, transdiagnostic sample), characteristics of trial participants (age, sex, diagnosis, severity of illness), type of intervention (content, intensity, duration, setting, parental involvement), measurement time points, type of outcome measure (instruments, blinded assessment), key findings (effect sizes), treatment fidelity and adherence checks, drop-out rates, study limitations, and funding sources.

### Risk of bias assessment

We evaluated the risk of bias in individual studies according to the Effective Public Health Practice Project (EPHPP) [36] recommendations on the domains selection bias, study design, confounders, blinding, data collection methods, and withdrawals and dropouts. Risk was quantified as weak, moderate, or strong. Studies without areas rated as weak were deemed as “strong”. One weak area led to a rating of “moderate” quality. Studies with two or more weak domains were classified as “weak”. There may also be selective reporting within studies, e.g. whether samples from the same group were truly independent, lack of reporting concerning adherence or blinding of raters. Regarding the risk of bias across studies, there is likely a high risk of publication bias considering that uncontrolled studies are easy to conduct.

### Summary measure and meta-analysis

The summary measure was the standardized mean difference (before and after the intervention). For the meta-analysis, we included studies reporting on ED psychopathology assessed with the EDE interview (EDE) or self-report questionnaire (EDE-Q) global score as their primary outcome. We calculated individual effect sizes using the pre-intervention SD [37]: $\mathrm{d}\  pr e=\frac{\mathrm{m}\  pos\mathrm{t}-\mathrm{m}\  pr\mathrm{e}}{\mathrm{SD}\  pr e.}$

We assumed an intra-study correlation of 0.5. One study [38] only reported t-test values, from which we calculated d [39]. According to Cohen, effect sizes of d < 0.5 were interpreted as small, 0.5 to 0.8 as medium and > 0.8 as large [40]. We accounted for differences in sample size by calculating the weighted mean effect sizes using the inverse variance weight according to Hedges and Olkin [39]. The overall effect size was calculated by dividing the sum of all weighted effect sizes by the sum of all weights. To assess significance, we calculated the standard error as $S\mathrm{E}=\sqrt{\frac{1}{\sum \mathrm{w}i}}$ and z scores as $z=\frac{\mathrm{d}\  mean-0.00}{\mathrm{SE}}$ . Z-scores above 1.96 were considered significant.

The confidence interval was defined as CI_95%_ = d_mean_ ± 1.96 SE.

To assess homogeneity, we used Cochran’s Q: $\sum \mathrm{w}i\ast \mathrm{d}{i}^2-\frac{\left(\sum \mathrm{w}i\ast \mathrm{d}i\right)2}{\sum \mathrm{w}i}$

As Cochran’s Q possesses insufficient power to detect true heterogeneity in small samples [41], we additionally calculated I^2^, which indicates the percentage of observed heterogeneity (I^2^ = 0.45 indicates 45% heterogeneity). An I^2^ of 25% was considered as low, 50% as moderate, and 75% as substantial heterogeneity [42].

## Results

### Study characteristics

**Fig. 1:**
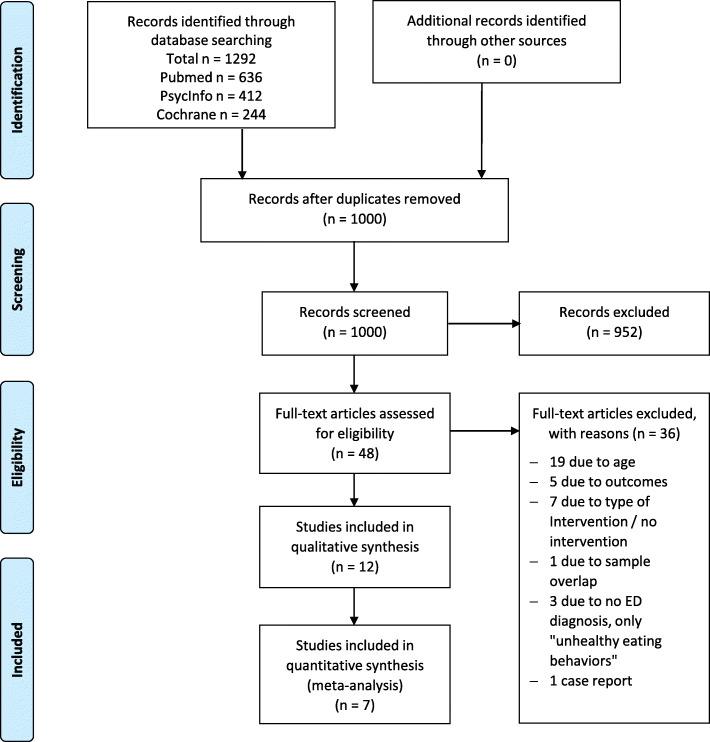
PRISMA Flow Diagram

The PRISMA flow diagram (Fig. 1) provides a detailed overview of the search and inclusion process [43]. A total of 1292 studies were identified, from which duplicated articles (*n* = 292) were removed. The remaining abstracts (*n* = 1000) were screened by two raters (JG, LH) to determine their relevance to this review. Nine hundred fifty-two studies were excluded according to both raters because they were deemed irrelevant. Two authors ( AB, JG) then independently screened the full text of the remaining articles (*n* = 48) and excluded 36 records that did not meet the inclusion criteria. Finally, 12 studies were included in the review.

Table 1 presents the study characteristics. The twelve studies included a total of 487 participants (female 97.3%/male 2.7%). Ten used a transdiagnostic sample, one a BN sample, and one sampled individuals with EDNOS. The age ranged from 11 to 21 years, with a mean of 15.6 years (SD = 0.81). Only Baudinet et al. (2020) and Timko et al. (2015) included boys (*n* = 13). Sample sizes ranged from 10 [46] to 131 patients [47]. Information on comorbidities and illness duration was not provided for all samples. On average, comorbid disorders were present in approximately 50%, although the highest reported comorbidities were 75 and 76.6%. Only the study by Fischer et al. provided information on non-suicidal self-injury (NSSI; 10 out of 10 patients) or previous suicide attempts (9 out of 10 patients). Accurso reported a mean illness duration of 6.43 months (SD = 3.38), while Johnson reported 1.9 years (SD = 1.55). Two studies observed inpatient treatment [50, 54], one combined day-care hospital and outpatient treatment [48], one observed day-care treatment [45], and eight examined outpatient treatment. Dropout rates ranged from 3.2 to 36.3%. The lowest dropout rate was from a single study group in Germany, comprising patients diagnosed with AN or BN [54], and the highest was found in a study with a standalone skills-based group intervention for BED/LOC [55]. Intervention durations ranged from 8 to 12 sessions over 3 months [55], to 77 days of combined treatment of day-care hospital and outpatient treatment [48].

**Table 1:** Efficacy of third-wave psychotherapeutic intervention for EDs in adolescents

Publication	Sample	Drop Outs	Time points	Intervention	Outcomes	Key findings^a^
Accurso et al. (2018) [44]	• Female only • Age 11–18 years (m = 15.5 ± 1.78 years) • AN (*n* = 11)	36.3%	EOT	• Outpatient • FBT plus DBT (ST) • 6 months	EDE subscales and global score, PEDE-Q, DERS, DTS, CSQ-8, HAQ, BMI	Significant reductions for BMI and PEDE-Q (not significant for ED patients), significant changes for DERS, DTS (patient and caregivers)
Baudinet et al. (2020) [45]	• 94.6% female, 5.4% male • Age 11–18 years (m = 15.02 ± 1.52 years) • AN-R (*n* = 109), AN-BP (*n* = 6), AAN (*n* = 7), EDNOS (*n* = 8)	26.7%	EOT	• Day-care/outpatient • Ro-DBT • m = 13.4 weeks	EDI-3, MFQ, SCS-R, ASQ, YSR, FFOCI-SF, SNYP-Y, TEPS, ERQ	Significant reductions in drive for thinness (EDI-3), depressive mood (MFQ), social connectedness (SCS-R), and emotional expressiveness (ERQ)
Fischer & Peterson (2015) [46]	• Female only • Age 14–17 years (m = 16.2 ± 1.03) • EDNOS (*n* = 10)	30%	EOT 6 months	• Outpatient • DBT • 6 months	EDE global score, frequency of NSSI and binge-purge behaviours, BMI, BDI	Significant reduction in EDE scores, NSSI, frequency of binge episodes and purging; at 6-month follow-up, 3 patients in remission
Johnston et al. (2015) [47]	• Female only • Age 12–17.5 years (m = 14.8 ± 1.5) • AN (*n* = 17), BN (*n* = 6), EDNOS (*n* = 28)	29%	EOT 3 months 6 months 12 months	• Outpatient • DBT (ST) plus MFT • m = 22.2 days (6 sessions ST á 45 min per week for 8 weeks)	EDE-Q global score, frequency of binge-purge behaviours, weight gain, BMI	Significant reduction in EDE-Q scores; significant increase in BMI, no changes in binge-purge behaviours; at 1-year follow-up full restoration in 64% of completers in menstruation and weight
Mazzeo et al. (2016) [38]	• Female only • Age 13–17 years (m = 15.4 ± 1.73) • BED/LOC (*n* = 45)	35.7%	EOT	• Outpatient • LIBER8 vs.2BFit • 8–12 sessions over 3 months	EDE-Q subscales and global score, EAH-C, EES-C	LIBER8: significant changes in EDE-Q subscales Eating and Shape Concern, Restraint and Global Score and on the EAH Negative Affect Scale. 2Bfit: significant reductions on all EDE-Q subscales and the Global Score and on the AAF and DEP subscales of the EES-C
Murray et al. (2015) [48]	• Female only • Age 14–17 years • (m = 15.7 ± 1.11 years) • BN (*n* = 40)	12.5%	EOT	• Day-care/Outpatient • DBT elements plus FBT • m = 77.2 days	EDE-Q subscales and global score, DERS subscales and global score, frequency of binge-purge behaviours, secretive eating frequency, BMI	Significant reductions in EDE-Q subscales Shape and Weight Concern and Global Score, significant reduction in DERS scale “access to emotion regulation strategies” and in frequency of binge episodes, purging and covert eating
Peterson et al. (2019) [49]	• Female only • Age 13–18 years • (m = 15.3 ± 1.64 years) • AN-R (*n* = 11), AN-BP (*n* = 2), AAN (*n* = 4), EDNOS (*n* = 1)	33.3%	EOT	• Outpatient • DBT (ST) plus FBT • 6 months	EDE-Q subscales and global score, CDI2, DBT-WCCL subscales, EBW, diary card	Significant reductions in EDE-Q subscale Restraint and Global Score, significant increase in adaptive skills and reduction in dysfunctional coping strategies measured with DBT-WCCL, significant reduction in CDI-2 and in OBE, significant increase in EBW
Salbach et al. (2007) [50]	• Female only • Age years (m = 16.0 ± 1.6 years) • AN-R (*n* = 17), AN-BP (*n* = 6), BN *n* = (8)	3.2%	EOT	• Inpatient • 12 weeks	EDI-2, SIAB-EX (frequency of binge-purge behaviours), SCL-90-R (depression scale and GSI), BMI	Significant reductions on all EDI-2 subscales except perfectionism, on SIAB domains frequency of binging and purging, avoiding calories, fasting, excessive sports, and use of laxatives; BMI increase in the AN group
Salbach-Andrae et al. (2008) [51]	• Female only • Age 12–18 years (m = 16.5 ± 1.0) • AN-R (*n* = 4), AN-BP (*n* = 2), BN (*n* = 6)	7%	EOT	• Outpatient • • 25 weeks	EDI-2, SIAB-EX (frequency of binge-purge behaviours), SCL-90-R (GSI), BMI	AN patients: at EOT 4 restricting type and 1 purging type fully remitted; significant increase BMI BN patients: at EOT 3 patient with BN fully remitted, 2 EDNOS, and 1 dropout Overall: significant reductions in all EDI subscales, food restriction, vomiting and binging, and GSI
Salbach-Andrae et al. (2009) [52]	• Female only • Age 12–21 years (m = 16.9 ± 1.7) • AN-R (*n* = 26), AN-BP (*n* = 11), BN (*n* = 13)	10%	EOT	• Outpatient • DBT vs. CBT vs. WCG • 25 weeks for CBT and DBT, 12 weeks for WCG	SIAB-EX (binge-purge behaviours, calorie restriction), EDI-2, GSI (SCL-90-R), BMI	at EOT 42.1% of CBT group, 37.5% of DBT group, and all patients of WCG still fulfilled eating disorder criteria CBT and DBT comparable improvements in calorie avoidance, meal frequency, current psychological distress and BMI
Schneider, et al. (2010) [50]	• Female only • Age 12.7–18.6 years (m = 16.3 ± 1.3) • AN-R (*n* = 27), AN-BP (*n* = 6), BN (n = 8)	18%	EOT	• Inpatient • DBT • m = 12 weeks	EDI-2, SIAB-EX (frequency of binge-purge behaviours, calorie restriction, irregular eating), GSI (SCL-90-R), BMI	At EOT 21 AN-R patients, 4 AN-BP patients, and 7 BN patients were fully remitted, Significant improvements in EDI-2 scales Drive for Thinness, Bulimia, Maturity Fears, and Interoceptive Awareness; significant improvements on SIAB domains frequency of binging and purging, avoiding calories, fasting, excessive sports; significant BMI increase
Timko et al. (2015) [53]	• 89% female, 11% male • Age 12–18 years (m = 14.0 ± 1.58) • AN/subthreshold AN (*n* = 47)	14.9%	EOT	• Outpatient • ASFT • 20 sessions over 24 weeks	EDE, BMI, remission status DERS (Non-Acceptance subscale), AFQ-Y, ABOS, AAQ, FamQ (Criticism and Emotional Over-involvement scale)	Significant weight increase and significant reduction on all EDE scales, remission full (48%), partial (29.8%) or none (21.3%), significant increase in parental acceptance (AAQ-2)

*2BFit* Weight management group, *AAQ-2* Acceptance and Action Questionnaire, *AFQ-Y* Action and Fusion Questionnaire-Youth, *ASFT* Acceptance-based Separated Family Treatment, *AN* Anorexia Nervosa, *AAN* Atypical Anorexia Nervosa, *AN-BP* Anorexia nervosa (binge-purging type), *AN-R* Anorexia nervosa (restrictive type) ASQ Attachment Style Questionnaire, *BDI* Beck Depression Inventory, *BED* Binge-eating disorder, *BMI* Body Mass Index, *BN* Bulimia Nervosa, *CDI-2* Child Depression Inventory-2, *CSQ-8* Client Satisfaction Questionnaire, *DBT-WCCL* DBT Ways of Coping Checklist, *DERS* Difficulties in Emotional Regulation Scale, *DEP* Depressive Symptoms, *DTS* Distress Tolerance Scale, *EAH-C* Eating in the Absence of Hunger Questionnaire for Children and Adolescents, *EBW* Expected body weight, *EDE* Eating Disorder Examination, *EDE-Q* Eating Disorder Examination-Questionnaire, *EDI* Eating Disorder Inventory, *EDI-2* Eating Disorder Inventory-2, *EDI-3* Eating Disorder Inventory-3, *EDNOS* Eating disorder not otherwise specified, *EES-C* Emotional Eating Scale-adapted for children, *EOT* End of treatment, *ERQ* Emotion Regulation Questionnaire, *FamQ* Family Questionnaire, *FBT* family-based treatment, *FFOCI-SF* Five-Factor Obsessive Compulsive Inventory-Short Form, *GSI* Global Severity Index, *HAQ* Helping Alliance Questionnaire, *IT* Individual therapy, *LIBER8* Linking Individuals Being Emotionally Real, *LOC* Loss of Control Eating, *M* mean, *MFT* Maudsley Family Therapy, *MFQ* Moods and Feelings Questionnaire, *NSSI* Non-Suicidal Self-Injury, *OBE* Objective Binge Eating, *PEDE-Q* Parent Eating Disorder Examination, *PVA* Parents Versus Anorexia scale, *SCL-90-R* Symptom Checklist-90-R, *SCS-R* Social Connectedness Scale-Revised, *SIAB* Structured Interview for Anorexic and Bulimic Disorders, *SIAB-EX* Structured Interview for Anorexic and Bulimic disorders for DSM-IV and ICD-10 for expert rating, *SNAP-Y* Schedule for Nonadaptive and Adaptive Personality for Youth, *ST* only skills training, *TEPS* Temporal Experience of Pleasure Scale, *WCG* waitlist control group, *YSR* Youth Self Report, ^a^all key findings based on completer analysis, none of the studies used an intention-to-treat analysis

Eleven studies assessed the effectiveness of DBT elements. Three of these studies developed a combination of DBT and FBT [44, 48, 49], one used a modified DBT-A skills-based group intervention [55], one studied radically open DBT [45], and one developed a combination of DBT and Maudsley-based family therapy [47]. Five studies evaluated ‘full-scale DBT’ treatment comprising all four modi (individual psychotherapy, skills group, telephone coaching, consultation team) [46, 50–52, 54]. Timko and colleagues (2015) developed Acceptance-based Separated Family Treatment (ASFT), a combination of ACT and FBT.

There were two RCTs, one with three arms (DBT-A vs. CBT vs. waitlist control) [52] and one with two arms (DBT-A-based skills group training (LIBER8) vs. weight management group (2BFit)), although the randomization procedure was only realized for 35 of the 45 patients [38]. Nine studies were uncontrolled. The majority of studies used only a pre- and post-treatment design without follow-up (*n* = 8); three studies measured six-month follow-up data [44, 46, 49] and one study included a 12-month follow-up [47]. Five studies were characterized as pilot studies [46–49, 54]. With the exception of the Salbach group, none of the other groups published or registered further studies following the pilot results.

### Risk of bias in individual studies

Assessments of overall study quality revealed that only one study was of moderate quality, while 11 out of the 12 included studies were classified as weak quality (see Table 2).

**Table 2:** Overall assessment of study quality according to EPHPP criteria

	Selection bias	Study design	Con-founders	Blinding	Data collection methods	Dropouts	Total
**Accurso et al. (2018)** **[**44**]**	Moderate	Weak	Weak	Weak	Moderate	Strong	Weak
**Baudinet et al. (2020)** **[**45**]**	Moderate	Weak	Weak	Weak	Strong	Moderate	Weak
**Fischer et al. (2015)** **[**46**]**	Moderate	Weak	Weak	Weak	Moderate	Moderate	Weak
**Johnston et al. (2015)** **[**47**]**	Moderate	Weak	Weak	Weak	Moderate	Moderate	Weak
**Mazzeo et al. (2016)** **[**38**]**	Moderate	Strong	Weak	Weak	Moderate	Moderate	Weak
**Murray et al. (2015)** **[**48**]**	Moderate	Weak	Weak	Weak	Strong	Strong	Weak
**Peterson et al. (2019)** **[**49**]**	Strong	Weak	Weak	Weak	Strong	Moderate	Weak
**Salbach et al. (2007)** **[**54**]**	Moderate	Weak	Weak	Weak	Moderate	Strong	Weak
**Salbach-Andrae et al. (2008)** **[**51**]**	Moderate	Weak	Weak	Weak	Moderate	Strong	Weak
**Salbach-Andrae et al. (2009)** **[**53**]**	Moderate	Strong	Weak	Moderate	Moderate	Strong	Moderate
**Schneider et al. (2010)** **[**50**]**	Moderate	Weak	Weak	Weak	Moderate	Strong	Weak
**Timko et al. (2015)** **[**53**]**	Strong	Weak	Weak	Weak	Moderate	Strong	Weak

### Risk of bias across studies

Due to the small number of included studies, we did not perform an analysis of publication bias [56]. We did not observe any selective reporting regarding outcomes.

### Meta-analytic findings

We found a moderate overall effect size of third-wave therapies on ED symptoms (d = − 0.67; z = − 6.99 C_95%_I = − 0.87 to − 0.47). Significant heterogeneity emerged (Cochran’s Q = 17.56 df = 6, critical value = 12.592; I^2^ = 0.65, indicating considerable heterogeneity), suggesting that the results were likely influenced by differences between studies. For an overview of effect sizes of all studies, please refer to Table 3.

**Table 3:** Overview of effect sizes of all included studies

**Studies included in the meta-analysis**		**N**	**Cohen’s d**	**Effect size**
**Accurso et al. (2019)** **[**44**]**	EDE Interview (global score)	11	−0.12	small
**Fischer et al. (2015)** **[**46**]**	EDE Interview (global score)	7	−0.63	moderate
**Johnston et al. (2015)** **[**47**]**	EDE-Q (global score)	33	−0.68	moderate
**Mazzeo et al. (2016)** **[**38**]**	EDE-Q (global score)	12	−0.50	moderate
**Murray et al. (2015)** **[**48**]**	EDE-Q (global score)	35	−1.53	large
**Peterson et al. (2019)** **[**49**]**	EDE-Q (global score)	12	−0.26	small
**Timko et al. (2015)** **[**53**]**	EDE Interview (global score)	32	−0.62	moderate
**Studies not included in the meta-analysis**		**N**	**Cohen’s d**	**Effect size**
**Baudinet et al. (2020)** **[**45**]**	EDI-3 (subscales)	105	−0.07 to − 0.39	small
**Salbach et al. (2007)** **[**54**]**	EDI-2	31	−0.02 to − 0.63	small – moderate
**Salbach-Andrae et al. (2008)** **[**51**]**	EDI-2 (subscales)	12	−0.42 to −3.03	small – large
**Salbach-Andrae et al. (2009)** **[**52**]**	prä-post EDI-2 (subscales)	16	−0.36 to −1.56	small –large
	SIAB (subscales)	16	−0.08 to −2.29	small – large
	DBT vs. WCG EDI-2 (subscales) ^a^	31	0.70 to 1.47	large
	SIAB (subscales) ^a^	31	1.44 to 1.85	moderate – large
**Schneider et al. (2010)** **[**50**]**	EDI-2 (subscales)^bc^	41	0.38 to 0.40	small
	SIAB (subscales)^c^	41	0.21 to 2.14	small – large

^a^ calculated from η^2^ in original publication ^b^ Authors only provided data for selected EDI subscales, ^c^ effect sizes from original publication due to unreported m and SD

### Qualitative synthesis

Study characteristics are summarized in Table 1. The qualitative analysis comprised all studies from the literature review and not only those included in the meta-analysis.

#### RCTs

We identified two RCTs. Salbach and colleagues (2009) compared DBT, CBT and a waitlist control group in a transdiagnostic sample (AN, BN). DBT and CBT did not differ, and were statistically more efficacious than waitlist control regarding remission rates, calorie avoidance, meal frequency, current psychological distress, and BMI (AN). Mazzeo et al. (2015) compared a skills-based group therapy (LIBER8) to a behaviour-based weight management group (2Bfit) in adolescents with binge and loss of control eating. There were significant reductions over time in eating disorder cognitions, dietary restraint and eating in response to negative affect, but no differences between treatment groups. The remaining studies in this review were uncontrolled.

#### Studies with DBT elements

One of the RCTs used DBT elements [55]. Accurso and colleagues (2018) combined family-based treatment with DBT skills training in patients with AN in community-based specialist clinics. Significant changes were observed for BMI, parent- and youth-reported Distress Tolerance Scale (DTS) scores, and Difficulties in Emotion Regulation Scale (DERS) scores. Changes in EDE-Q scores were significant according to the parent-reported but not the youth-reported version. Fisher and colleagues (2015) examined a sample of adolescents with EDNOS, binge eating and NSSI, and found significant reductions in EDE scores, frequency of binge episodes and purging, and NSSI at the end of treatment, which were stable at 6-month follow-up. A pilot study by Johnston et al. (2015) examined Maudsley family therapy with DBT skills training in a transdiagnostic sample, finding significant reductions in EDE-Q scores, a significant increase in BMI, but no effect on binge-purge behaviours from pre-treatment to discharge and 3-, 6-, and 12-month follow-up. At the 1-year follow-up, 65% of the sample were weight-restored and menstruating normally. Murray et al. (2015) sampled adolescents with BN in an open pilot trial to investigate the efficacy of a program integrating family-based treatment and DBT. The authors reported significant reductions in EDE-Q scores (subscales Shape and Weight Concern, Global Score), improvements in access to emotion regulation strategies (DERS) and binge-purging episodes at discharge. Another uncontrolled trial combined family-based treatment and DBT skills training in patients with restrictive EDs [49]. Significant reductions in EDE-Q scores (restraint eating, global score) and depression emerged in completers. The DBT Ways of Coping Checklist showed a significant increase in adaptive skills use and decrease in the use of dysfunctional coping strategies. Additionally, a significant decrease in binge eating and increase in percent expected body weight were reported. Baudinet and colleagues (2020) combined elements of individual, family, and group therapy, meal support, and education support in an intensive day-treatment program conducted from Monday to Friday. The group program consists of radical open dialectical behaviour therapy (RO-DBT, 2.5 h), with CBT (1.5 h), cognitive remediation treatment (45 min), and art therapy (1 h). The uncontrolled pre-post design showed significant improvements regarding drive for thinness, depressive mood, social connectedness and emotional expressiveness.

#### Full-scale DBT treatment

One of the RCTs implemented a full-scale DBT treatment (see above for description) [52]. Salbach et al. (2007) adapted DBT-A for an inpatient sample with AN or BN and observed significant reductions in most EDI-2 subscale scores and a BMI increase for AN-R/AN-BP. The SIAB showed significant reductions on frequency of binging and purging (AN-BP, BN), avoiding calorie intake, fasting, excessive sports, and use of laxatives. In a case series, the authors found a significant reduction on all EDI-2 subscale scores and the global severity index (GSI). All patients showed significant reductions in food restriction, whereas patients with AN-BP and BN additionally showed a significant reduction in frequency of vomiting / binge-eating [51]. Finally, an inpatient study by Schneider et al. (2010) reported significant reductions on the EDI-2 subscales Drive for Thinness, Bulimia, Maturity Fears and Interoceptive Awareness, on the SIAB domains frequency of binging and purging, avoiding calories, fasting, and excessive sports, and a significant BMI (AN) increase.

#### Act

An open trial of Acceptance-based Separated Family Treatment (ASFT) for adolescents with AN from Timko and colleagues (2015) revealed a significant weight increase, significant reduction on all EDE scales, and a significant increase in acceptance of emotions.

## Discussion

This review with meta-analysis examined the empirical evidence of third-wave therapies for the treatment of EDs in adolescents (adaptation and efficacy). We identified a total of two RCTs and ten uncontrolled pre-post studies. Our meta-analysis of seven pre-post studies using the EDE as an outcome measure found an overall moderate effect size (d = − 0.67). However, since these findings are based on uncontrolled studies, it is impossible to know to what extent the effect is caused by the therapy or by extraneous variables such as unspecific treatment effects, spontaneous recovery, or regression to the mean [57].

The two RCTs revealed symptom improvements over time: Salbach et al. (2009) showed that DBT was more efficacious than waitlist control in terms of calorie restriction, irregular eating, and current psychological distress as well as BMI, although it was not superior to an active control group. Mazzeo and colleagues (2016) also found significant but comparable improvements in both a third-wave group and active control group for dietary restraint, eating disorder cognitions, and eating in response to negative affect. Overall, the third-wave treatment resulted in moderate to large improvements in eating disorder symptoms in all but two studies [44, 49]. These results are consistent with the effects of non-third-wave outpatient treatments for ED in adolescents (AN: CBT (d = − 0.83) [14], FBT (d = − 0.85), AFT (d = − 0.84) [58]; BN: CBT (d = − 0.83), PDT (d = − 0.98) [20], CBT-A (d = − 1.2), FBT-BN (d = − 1.3) [19]).

As expected, there is a considerable difference between the number of studies investigating the efficacy and/or effectiveness of third-wave ED treatment in adolescent versus adult samples. For adults, Linardon and colleagues (2017) identified 13 RCTs and 14 uncontrolled studies, while we found only two RCTs and nine uncontrolled studies for childhood and adolescence. Effect sizes in adults were larger (overall third-wave d = − 1.07 and DBT d = − 1.15). The study quality was also higher in the adult trials, with most studies being of moderate quality, whereas studies in childhood and adolescence were of predominantly weak quality.

The informative value of the studies on children and adolescents was hindered by several factors:

- The uncontrolled study design limits the informative value regarding therapy efficacy, since the influence of extraneous factors cannot be ruled out.
- Sample sizes were small and did not allow for the analysis of possible confounders.
- The lack of blinded outcome assessments is a substantial limitation in terms of the reliability.
- Due to a lack of follow-up assessment, we have no information regarding the long-term effectiveness of the interventions.
- There are no clear replication studies, since the sample composition (AN, BN, BED, EDNOS) differed for each trial.

Although data collection methods were rated as strong according to the EPHPP criteria laid out by Thomas et al. (2004), this rating only pertains to the instruments’ reliability and validity. It does not take into account the need for blinded outcome assessments or the higher informational content of clinical interviews compared to questionnaires. Blinded outcome assessments are critical for ruling out bias and therefore invaluable for high-quality trials. Unfortunately, most studies in this review opted to use the EDE questionnaire instead of the available clinical EDE interview, and those using the interview did not employ blinded clinicians as interviewers. Furthermore, it is noteworthy that despite third-wave therapies focusing heavily on emotion regulation as their mechanism of change, only two studies included a measure of emotion regulation in their outcomes [44, 53]. It remains unclear to what degree the reported symptom improvements were related to emotion regulation. It is possible that the effects were mainly due to therapeutic strategies that are not specific for third-wave therapies but instead rely on treatment components of CBT or FBT.

All of the studies used either DBT or ACT, although a wide variety of treatments are counted as third-wave interventions (ACT, CFT, DBT, MBI and ST). Thus, it is not possible to conclusively assess the overall efficacy of third-wave treatments.

Almost all studies (*n* = 11) used DBT elements in their protocol, making DBT the most widely studied third-wave therapy for EDs. Surprisingly, only one study investigated a modified version of RO-DBT. This treatment was developed to target maladaptive overcontrol behaviour, a proposed core difficulty of restrictive eating disorders [45]. As there was only one study with RO-DBT and due to the low study quality, we cannot make any statement about the effectiveness in comparison to standard DBT. However, the results on DBT in ED treatment should be interpreted with caution due to two critical points:

- Diagnoses under the ED umbrella are highly heterogeneous, with different aetiologies, clinical presentations, and disorder mechanisms. It is therefore reasonable to assume differential effects of any given treatment on AN, BN and BED. Indications of this can be seen in Linardon et al. (2017). However, since many of the studies in children and adolescents were based on transdiagnostic samples (*n* = 7), and the sample sizes were too small to conduct sub-analyses for each diagnosis, the results largely refer to the efficacy of DBT on the general category of ED. This can obscure ED-specific effects.
- The second major critical aspect pertains to the question: What are the minimum requirements regarding treatment composition, treatment intensity, and qualifications for the therapists to call an intervention a DBT intervention? Among the studies in this review, there was considerable variation in terms of the dosage of DBT. For example, Accurso et al. (2018) included only four skills training sessions, while the main part of the treatment consisted of FBT. Thus, the study evaluated the efficacy/effectiveness of FBT supplanted with DBT elements, not DBT as it is conceptualized by Linehan [59]. Baudinet et al. also only used the group skills training component of RO-DBT [45]. This was an issue in six of the DBT studies. Only five of the eleven DBT studies actually comprised all four modi [60] of the DBT treatment. Four of these studies originated from the same research group of Salbach and colleagues [50–52, 54]. It cannot be conclusively ascertained whether there was an overlap between samples. Incidentally, these studies also reported the largest effect sizes, which may be due to the more complete implementation of DBT. We cannot answer this question, because a comparison with “low DBT dosage” is hindered by the different sample compositions. Independent replication studies are needed to assess dosage effects of DBT. Furthermore, the majority of the DBT studies (*n* = 7) provided no information regarding therapists’ qualifications. Only one group used clinical psychologists for its studies, and only two groups confirmed that their therapist had completed specialist DBT-A training. Lastly, we did not find any detailed information regarding treatment fidelity. Some authors mentioned supervision as a quality control measure, but none mentioned or reported data regarding adherence checks to ensure that it was a true DBT intervention.

ACT has only been investigated in one uncontrolled pre-post study. More precisely, ACT principles were combined with elements of FBT and showed moderate effect sizes. For this as well as the aforementioned reasons, no clear assertions can be made regarding the extent to which ACT is efficacious for EDs in adolescence.

### What are the implications for clinical/practical work and research?

There is mounting evidence that alternative treatments for EDs across the lifespan are needed. Cowdrey and Waller [61] stated that practitioners in adulthood are increasingly applying third-wave therapies to adults with ED, suggesting that patients do not sufficiently benefit from existing treatment options. The same seems to be true for adolescence. FBT as the single most well-researched and effective treatment depends upon commitment from the entire family system. This can be a considerable obstacle in many families, especially during adolescence. It is therefore imperative to at least have an effective alternative treatment that can integrate the family but can also function without support from all members of the system. Long-term follow-ups show that the superiority of FBT versus active control treatments disappears. However, AFT, which supplements DBT components, was equally as effective and in some areas slightly superior at a 4-year follow-up [62].

Despite the clinical demand, there is a lack of high-quality research on the efficacy of specific third-wave interventions for adolescents with ED. None of the existing interventions meet the criteria for an empirically supported treatment. The existing studies only allow the conclusion that third-wave interventions are possibly efficacious and constitute valuable alternative treatment options. However, the majority of studies in our review had very low dropout rates (> 18%). This is a substantial advantage over non-third-wave trials, with dropouts of around 30% [8].

We agree with the assessment by Linardon et al. (2017) that conducting large-scale RCTs can be prohibitive for many institutions and practitioners who may already work with third-wave interventions, and thus (single) case studies akin to the study by Salbach et al. (2008) may be a good starting point. This format is highly clinically relevant in terms of feasibility [63] and can provide valuable information for researchers planning to investigate this treatment through an expensive RCT as well as for agencies which are deciding on whether to fund such a trial [21]. Furthermore, future research on third-wave treatments coming from clinical practice could reduce the barriers to implementation and dissemination for other practitioners, ensuring that clients receive the best possible care. An RCT could then look beyond simple measures of efficacy by confirming specific treatment effects as well as assessing mechanisms of therapeutic change, the dosage of treatment required to be effective [64], and predictors of non-response [65].

### Limitations and strengths

The primary limitation is that the empirical evidence base comprises only a small number of studies, including only two RCTs. Our meta-analysis was based mostly on transdiagnostic samples. This limits the informational content, since each ED diagnosis comes with a different aetiology and different sustaining factors, rendering it highly likely that different therapeutic strategies are needed to change patients’ dysfunctional emotion regulation. We only included studies published in English in peer-reviewed journals. In addition to the publication bias, a bias due to excluded non-English-language publications may also have occurred. Lastly, since there is no comprehensive list of third-wave therapies, no conclusive evaluation of its efficacy can be made.

## Conclusion

This review with meta-analysis was the first to evaluate the adaptation and efficacy of third-wave therapies for the treatment of ED in adolescents. The main limitation of the empirical database is that almost all studies used DBT, while other third-wave treatments lacked empirical evaluation. Despite promising evidence of a beneficial impact of DBT, none of the treatments meet the criteria for an empirically supported treatment. Due to high relapse rates, there is an urgent need for further high-quality research into alternative ED treatments. Effective early interventions in adolescence might prevent chronification and help both patients and the healthcare system in the long run.

## Supplementary Information

**Additional file 1:.** PRISMA 2009 Checklist.

**Additional file 2:.** Review Protocol.

**Additional file 3:.** Search strategies.

## Data Availability

PRISMA checklist, review protocol and search strategies available as supplemental material.

## Abbreviations

ACT: Acceptance and commitment therapy
AFT: Adolescent-focused therapy
AN: Anorexia nervosa
AN-BP: Anorexia nervosa (binge-purging type)
AN-R: Anorexia nervosa (restrictive type)
ASFT: Acceptance-based separated family treatment
BED/LOC: Binge eating disorder / loss of control
BMI: Body mass index
BN: Bulimia nervosa
CBT: Cognitive behaviour therapy
CBT-A: cognitive-behavioral therapy adapted for adolescents
CBT-E: Enhanced cognitive behaviour therapy
CFT: Compassion-focused therapy
DBT: Dialectical behaviour therapy
DBT-A: Dialectical behaviour therapy for adolescents
DERS: Difficulties in Emotion Regulation Scale
DP: Day-patient treatment
DSM-IV: Diagnostic and statistical manual of mental disorders, 4th edition
DTS: Distress tolerance scale
ED: Eating disorder
EDE: Eating disorder examination
EDE-Q: Eating disorder examination-questionnaire
EDI-2: Eating disorder inventory-2
EDI-3: Eating disorder inventory-3
EDNOS: Eating disorder not otherwise specified
EPHPP: Effective public health practice project
FBT: Family-based therapy
GSI: Global severity index
ICD-10: International classification of diseases, 10th edition
IP: Inpatient treatment
MBI: Mindfulness-based interventions
NSSI: Non-suicidal self-injury
PICOS: Acronym for patients – intervention – comparator – outcomes – study design
PRISMA: Preferred reporting items for systematic reviews and meta-analyses
RCT: Randomized controlled trial
RO-DBT: Radical open dialectical behaviour therapy
SIAB-EX: Structured interview for anorexic and bulimic disorders for DSM-IV and ICD-10
ST: Schema therapy
