# Supplementary material for: Third-wave interventions for eating disorders in adolescence – systematic review with meta-analysis
Source: Borderline Personal Disord Emot Dysregul. 2021 Jun 14;8:20. doi: 10.1186/s40479-021-00158-6 (PMC8201936; doi:10.1186/s40479-021-00158-6)
Supplement: Supplementary file 2 — Additional file 2:. Review Protocol. [file 40479_2021_158_MOESM2_ESM.docx]

**- Review Protocol -**

*Third-Wave Psychotherapy Interventions for the Treatment of Eating Disorders in Adolescence – Systematic Review with Meta-Analysis*

**Review Question**

1) Which third-wave treatments have been adapted for *Eating Disorders (*EDs) in adolescents?

2) What is the empirical evidence regarding their efficacy, and are they comparable to specialized treatments (e.g. cognitive behaviour therapy, family-based therapy) of EDs in this age group?

**Searches**

We will search the following electronic bibliographic databases: PubMed, PsycINFO, The Cochrane Library (Cochrane Database of Systematic Reviews) using the following search terms:

1. third wave OR dialectical behavior therapy OR dialectical behaviour therapy OR dialectic behavioral therapy OR dialectic behavioural therapy OR DBT OR mindful* OR acceptance OR schema therapy OR compassio*

2. eating disorder OR bulimi* OR anorexi* OR binge OR EDNOS

3. adolesc* OR teen* OR youth OR children OR childhood OR pediatric

**Types of study to be included**

This review included randomized controlled trials and pre-post studies published in English in a peer-reviewed journal up until 21st November 2019

**Condition or domain being studied**

EDs have a high morbidity and mortality, and come with a significant personal and economic burden. Although substantial progress has been made in specialized treatments for EDs in adolescents, there is still room for improvement regarding treatment retention, outcomes and dropout rates. Third-wave therapies have the potential to be an alternative treatment for EDs. We review the empirical evidence on third-wave interventions for adolescents with EDs, and conduct a meta-analysis.

**Participants/Population**

We will consider samples comprising females aged 12-21 years with an ED diagnosis (anorexia nervosa(AN), bulimia nervosa (BN), binge eating disorder, eating disorder not otherwise specified (EDNOS). If a study sample extends beyond that age range, the publication needs to report results for the adolescent subgroup only in order to be included.

**Intervention(s), exposure(s)**

This review will be limited to studies investigating the efficacy of third-wave psychological interventions, i.e. acceptance and commitment therapy (ACT), compassion-focused therapy (CFT), dialectical behavior therapy (DBT), mindfulness-based interventions (MBI) and schema therapy (ST).

**Comparator(s) / control**

Presence of a comparison / control group will be not required for inclusion in the review. We also consider pre-post studies with no control group.

**Main outcome(s)**

Efficacy of the interventions has to be evaluate according to the Eating Disorder Examination (EDE) or Eating Disorder Examination-Questionnaire (EDE-Q), the Eating Disorder Inventory-2 (EDI) bulimia scale or the Structured Interview for Anorexic and Bulimic Disorders for DSM-IV and ICD-10 (SIAB-EX).

**Additional outcome(s)**

No additional outcome(s) will be assessed.

**Risk of bias (quality) assessment**

We will evaluate the risk of bias in individual studies according to the Effective Public Health Practice Project (EPHPP) recommendations on the domains selection bias, study design, confounders, blinding, data collection methods, and withdrawals and dropouts. Risk will be quantified as weak, moderate or strong. Studies without areas rated as weak will be deem as “strong”. One weak area will led to a rating of “moderate” quality. Studies with two or more weak domains will classified as “weak”. Regarding the risk of bias across studies, we will look at publication bias and selective reporting within studies, e.g. whether samples from the same group were truly independent, lack of reporting concerning adherence or blinding of raters.

**Strategy for data synthesis**

For the meta-analysis, we will include studies reporting on ED psychopathology assessed with the EDE interview (EDE) or self-report questionnaire (EDE-Q) global score as their primary outcome. We will calculated individual effect sizes using the pre-intervention

SD: $d pre= \frac{m post-m pre}{\mathrm{SD}pre}$

We will calculate d for studies only reported t-test values. When we aggregate the results, we will account for differences in sample size by calculating a weighted mean effect size [34]. To assess significance, we will calculate the standard error as $SE=\sqrt{\frac{1}{\sum wi}}$ and z scores as $z= \frac{d mean-0.00}{\mathrm{SE}}$ . Z-scores above 1.96 will be considered significant.

The confidence interval will be defined as CI_95%_ = d_mean_ ±1.96 SE.

To assess homogeneity, we will use Cochran’s Q: $\sum wi*di^{2}-\frac{(\sum wi*di)^{2}}{\sum wi}$

As Cochran’s Q possesses insufficient power to detect true heterogeneity in small samples, we will additionally calculate I², which indicates the percentage of observed heterogeneity. An I² of 25% will considered as low, 50% as moderate and 75% as substantial heterogeneity.

**Analysis for subgroups or subsets**

No analysis of subgroups or subsets are planned.
