# Supplementary material for: Third-wave interventions for eating disorders in adolescence – systematic review with meta-analysis
Source: Borderline Personal Disord Emot Dysregul. 2021 Jun 14;8:20. doi: 10.1186/s40479-021-00158-6 (PMC8201936; doi:10.1186/s40479-021-00158-6)
Supplement: Supplementary file 3 — Additional file 3:. Search strategies. [file 40479_2021_158_MOESM3_ESM.docx]

**Supplement 3: Search strategies**

**Pubmed**

(("third wave") OR (dialectical behavior therapy) OR (dialectical behaviour therapy) OR (dialectic behavioral therapy) OR (dialectic behavioural therapy) OR DBT OR mindful* OR acceptance OR schema therapy OR compassio*) AND ((eating disorder) OR bulimi* OR anorexi* OR binge OR EDNOS) AND (adolesc* OR teen* OR youth OR children OR childhood OR pediatric)

**PsycInfo**

((("third wave" OR "Dialectical behavior therapy" OR "Dialectical behaviour therapy" OR "Dialectic behavioral therapy" OR "Dialectic behavioural therapy" OR DBT OR mindful* OR acceptance OR "Schema therapy" OR compassio*) AND rtype.exact("Peer Reviewed Journal" OR "Journal Article" OR "Journal")) AND rtype.exact("Peer Reviewed Journal" OR "Journal Article" OR "Journal")) AND ((("eating disorder" OR anorexi* OR bulimi* OR binge OR EDNOS) AND rtype.exact("Peer Reviewed Journal" OR "Journal Article" OR "Journal")) AND rtype.exact("Peer Reviewed Journal" OR "Journal Article" OR "Journal")) AND (((adolesc* OR teen* OR youth OR children OR childhood OR pediatric) AND rtype.exact("Peer Reviewed Journal" OR "Journal Article" OR "Journal")) AND rtype.exact("Peer Reviewed Journal" OR "Journal Article" OR "Journal"))

**Cochrane**

|  | **Search Terms** |
| --- | --- |
| #1 | ("third wave") (Word variations have been searched) |
| #2 | (dbt) OR (dialectical behavior therapy) OR (dialectical behaviour therapy) OR (dialectic behavioral therapy) OR (dialectic behavioural therapy) (Word variations have been searched) |
| #3 | (mindful*) OR (acceptance) OR (compassio*) OR (schema therapy) |
| #4 | ("eating disorder") OR (anorexi*) OR (bulimi*) OR (binge) OR (EDNOS) |
| #5 | (adolesc*) OR (teen*) OR (youth*) OR (children) OR (childhood) (Word variations have been searched) |
| #6 | (pediatric) (Word variations have been searched) |
| #7 | (#1 OR #2 OR #3) AND #4 AND (#5 OR #6) |
